# Surviving Alpine Winters Without Dehydration: Physiological and Transcriptomic Insights into the Freeze Avoidance of *Megastigmus sabinae* Xu et He (Hymenoptera: Torymidae)

**DOI:** 10.3390/insects17090889

**Published:** 2026-08-25

**Authors:** Dong Lv, Erwen Xu, Bin Chen, Hu Zhao, Rong Zhou, Hang Xiang, Na Wei, Han Xu, Min Chen

**Affiliations:** 1 Gansu Province Academy of Qilian Water Resource Conservation Forests Research Institute, Zhangye 734000, China; 2 Beijing Key Laboratory for Forest Pest Control, Beijing Forestry University, Beijing 100083, China

**Keywords:** seed wasp, cold hardiness, supercooling point, aquaporin, cold acclimation

## Abstract

*Megastigmus sabinae* Xu et He (Hymenoptera: Torymidae) is an oligophagous seed pest overwintering within *Juniperus przewalskii* cones in the alpine Qilian Mountains. Understanding its cold adaptation is critical for forest pest management. To investigate its overwintering mechanisms, we monitored field temperatures and larval development. Subsequently, larvae were subjected to a series of low-temperature treatments under laboratory conditions. We then assessed survival rates, supercooling points (SCPs), free water content, and the transcriptomic profiles of water-regulatory genes. Field data revealed that larvae enter a quiescent state when ambient temperatures drop below 5 °C. Laboratory acclimation at 0 °C significantly improved larval survival at −10 °C and markedly depressed the SCP by approximately 6 °C. Notably, free water content remained completely stable across all temperature treatments. Consistent with this dehydration-independent phenotype, transcriptomic analysis showed that the majority of 17 aquaporins (AQPs) and a *Capa* receptor gene lacked differential expression. However, two specific AQP genes (TIP1-1 and AQPN) were significantly upregulated. Collectively, *M. sabinae* larvae employ a freeze-avoidance strategy driven by dehydration-independent SCP depression and highly targeted transcriptional adjustments of specific water channels.

## Simple Summary

The seed wasp *Megastigmus sabinae* is a destructive insect that damages the seeds of the important evergreen tree *Juniperus przewalskii* in high mountain forests. Therefore, it is necessary to understand how this pest survives harsh, freezing winters. Our study aimed to uncover the cold survival strategies of the wasp larvae, which hide inside tree cones. We found that the larvae enter a simple resting state when temperatures drop. Surprisingly, unlike many overwintering insects that lose body water to prevent ice damage, these larvae keep their body water levels completely stable. Instead, brief exposure to cold weather helps them naturally lower the exact temperature at which their internal liquids freeze. By examining their genes, we discovered they activate only a few specific genes that control water movement, rather than changing their entire water system. We conclude that this insect uses a highly unusual method to avoid freezing without drying out.

## 1. Introduction

*Megastigmus sabinae* Xu et He (Hymenoptera: Torymidae) is an oligophagous pest that damages the seed kernels within the cones of *Juniperus przewalskii* Kom. It is mainly distributed in the Qilian Mountains of China. The larvae of *M. sabinae* bore into and feed on the endosperm of the cones, causing the damaged seeds to lose their viability and severely threatening the reproduction and regeneration of *J. przewalskii* populations [1,2]. In some forest areas, the infestation rate exceeds 90% [3]. The larvae overwinter as second- or third-instar larvae inside the cones and resume development in mid-March of the following year, demonstrating strong adaptability to the low-temperature environments of the plateau region [4].

Cold hardiness is often closely linked to the minimum environmental temperature of species’ habitats [5,6]. Organisms have evolved two main overwintering strategies for cold tolerance: freeze avoidance, which prevents internal ice formation by depressing the freezing point or supercooling point (SCP) of body fluids, and freeze tolerance, which involves tolerating extracellular ice formation [7]. Most insects are freeze-avoidant [8], relying on significantly depressing the SCP, eliminating ice nucleators in their bodies, and accumulating non-colligative antifreeze proteins, polyhydric alcohols (polyols), sugars, and other low-molecular-weight cryoprotectants to maintain their body fluids in a supercooled state in subzero environments (−15 °C and below) [8,9,10]. Because most insects are unable to survive internal ice formation, the majority of studies of overwintering physiology in this group have focused on measuring the SCP [11,12,13].

The ability of insects to maintain ion and water balance under low-temperature stress is critical for their survival [14,15,16]. When the temperature drops below the supercooling point, the freezing of body fluids causes lethal damage [17]. Therefore, water management is a core component of cold hardiness mechanisms. Aquaporins (AQPs) are transmembrane channel proteins responsible for the directional transport of water and small-molecule solutes such as glycerol. They play essential roles in regulating osmotic balance, cell volume, and the transmembrane distribution of cryoprotective substances [18,19]. In arthropods, the AQP family is functionally divided into several subfamilies, including DRIP (water-specific), PRIP, aquaglyceroporins (Eglp, RPIP), BIB, and the atypical AQP12L [20,21]. Structurally, AQPs assemble as tetramers. Each monomer comprises six transmembrane helices and two conserved NPA motifs, utilizing an aromatic/arginine (ar/R) selectivity filter to control the size and hydrophobicity of the transported substrates [22,23]. Previous studies have found that low temperature or dehydration can induce the upregulation of specific AQP genes in insects, promoting intracellular water efflux or the influx of cryoprotective solutes, thereby enhancing cold hardiness [24,25,26]. Furthermore, neuropeptides and their receptors also participate in the maintenance of ion and water homeostasis in insects. In *Drosophila*, *Capa* neuropeptide signaling has been demonstrated to play a crucial role in resisting chill coma and maintaining ion balance during cold exposure [27]. However, the low-temperature responses of AQPs and the neuropeptide *Capa* receptor gene in high-elevation overwintering insects remain to be investigated.

Although the larvae of *M. sabinae* overwinter for several months within the cones, the specific cold hardiness strategies of this species remain unclear. Furthermore, the impact of cold acclimation on tolerance to extreme low temperatures, as well as the transcriptional responses of AQPs and neuropeptide *Capa* receptor genes during low-temperature adaptation, have yet to be elucidated. Therefore, focusing on a high-altitude population from the Qilian Mountains, this study systematically investigated the relationship between larval development and habitat temperature. We measured survival rates, supercooling points (SCPs), and body water content under various low-temperature and cold acclimation treatments. Additionally, we analyzed the expression profiles of AQP and neuropeptide *Capa* receptor genes using transcriptomic and qRT-PCR analyses. Ultimately, this research aims to reveal the physiological and molecular mechanisms of low-temperature adaptation in *M. sabinae*.

## 2. Materials and Methods

### 2.1. Insect Collection and Habitat Temperature

Larvae of *M. sabinae* were collected from the Toutan area (38°32′4″ N, 100°15′50″ E; elevation 2920 m) in Sunan Yugur Autonomous County, Zhangye City, Gansu Province, China. Branches bearing current-year cones of *J. przewalskii* were collected and transported to the laboratory. To track development, infested branches were collected monthly from August 2023 to July 2024, and larvae were extracted from the cones on approximately the 15th of each month. During this period, daily mean ambient temperatures (*T_i_*) were recorded by a local meteorological station at a standard measurement height of 1.5 m. The monthly mean temperature (*T_m_*) was calculated using the following equation: $T_{m}=\;\frac{\sum_{i=1}^{n}T_{i}}{n}$ where *T_i_* is the daily mean temperature on the *i*-th day of the month, and *n* is the total number of days in that month.

For all other laboratory experiments (including low-temperature exposure, supercooling point measurements, and transcriptomic analyses), branch samples were collected between October and December 2025, prior to the assays. To prevent desiccation, the cut ends of the branches were inserted into moistened floral foam. Larvae were subsequently extracted by dissecting the cones immediately prior to each experiment.

### 2.2. Measurement of Larval Mandible Width

The mandible width (defined as the maximum distance between the outer edges of the mandibles) of *M. sabinae* larvae was measured using a Leica M205 FA stereomicroscope (Leica Microsystems, Wetzlar, Germany).

### 2.3. Effect of Low-Temperature on the Survival Rate of M. sabinae Larvae

#### 2.3.1. Constant Low-Temperature Exposure at Varying Durations

Branches were placed in environmental chambers set to 20 °C, 10 °C, 0 °C, −10 °C, and −20 °C. Cones were dissected to extract *M. sabinae* larvae, and their survival was evaluated after 6, 12, 24, 48, 96 h and 7 d of exposure. For each temperature and time point, survival was assessed using 30 larvae. Larvae were gently prodded on the head with forceps, and those showing no behavioral response were classified as dead. Each treatment was replicated five times.

#### 2.3.2. Effect of Cold Acclimation on Larval Survival Under Extreme Low Temperature

Four different pre-treatments were applied for 7 days: constant temperatures of 20 °C, 10 °C, and 0 °C, and a fluctuating thermal regime at 0 °C (FTR 0 °C, 0 ± 5 °C). The FTR 0 °C treatment consisted of sequential 8 h exposures to −5 °C, 0 °C, and 5 °C (totaling 24 h per cycle). After preconditioning, the branches were immediately transferred to −10 °C. Cones were dissected, and larval survival was assessed at 6, 12, 24, 48, and 96 h of exposure. For each treatment, the survival rate of 30 larvae was recorded, with five independent replicates per treatment.

### 2.4. Effect of Cold Acclimation on the Supercooling Point and Body Water Content of M. sabinae Larvae

Five different pre-treatments were applied for 7 days: constant temperatures of 20 °C, 10 °C, 0 °C, and −10 °C, and a fluctuating thermal regime at 0 °C (FTR 0 °C, 0 ± 5 °C). The FTR 0 °C treatment was identical to that described in Section 2.3.2. Following preconditioning, the supercooling point (SCP) of the larvae was measured. Specifically, larvae were extracted from the cones and individually placed into 200 μL glass inserts, which were then housed within 1.5 mL sample vials. The vials were transferred from room temperature directly to a −80 °C environment. The cooling process was continuously monitored, and the SCP (indicated by the crystallization exotherm) was recorded using a USB single-channel K-type thermocouple thermometer (Dajia Sensor Technology Co., Ltd., Dongguan, China). Twenty larvae were evaluated per temperature treatment.

To determine the larval body water content, batches of 100 larvae from each treatment group were accurately weighed after the 7-day treatment using a microbalance (precision: 0.01 mg) to obtain the fresh weight (FW). The larvae were then dried in an oven at 60 °C for 48 h and re-weighed to obtain the dry weight (DW). The percentage of free water content (FC) was calculated using the following equation: FC = [(FW − DW)/FW] × 100%. This measurement was performed with four biological replicates per treatment.

### 2.5. Effects of Low-Temperature Exposure on the Expression of Aquaporin and Neuropeptide Capa Receptor Genes in M. sabinae Larvae

#### 2.5.1. Sample Preparation and Cold Treatments

Larvae were subjected to three constant temperature treatments: 20 °C, 10 °C, and 0 °C for 7 d. Five biological replicates were established for each treatment. Following exposure, the larvae were immediately flash-frozen in liquid nitrogen and stored at −80 °C for subsequent analyses.

#### 2.5.2. RNA Extraction and Transcriptomic Analysis

For each replicate, 20 s-instar larvae were ground into a fine powder in liquid nitrogen within a 1.5 mL microcentrifuge tube. Total RNA was extracted using the TRIzol reagent (Invitrogen, Waltham, MA, USA) strictly following the manufacturer’s protocol. RNA integrity was preliminarily assessed via 1% agarose gel electrophoresis, while RNA concentration and purity were quantified using a NanoDrop spectrophotometer (NanoDrop Technologies, Wilmington, DE, USA). Only RNA samples meeting the quality criteria of an A260/A280 ratio between 1.8 and 2.0, and an A260/A230 ratio greater than 2.0 were retained. The qualified RNA samples were preserved on dry ice and shipped to Shanghai Majorbio Bio-pharm Technology Co., Ltd. (Shanghai, China) for transcriptomic sequencing on an Illumina platform. The downstream bioinformatic analyses of the transcriptomic data were performed according to the methods described by Lu et al., 2023 [28].

#### 2.5.3. qRT-PCR Validation of Differentially Expressed Genes

To validate the transcriptomic expression profiles of aquaporin and neuropeptide *Capa* receptor genes), three genes significantly upregulated under cold stress were selected for quantitative real-time PCR (qRT-PCR) analysis. Specific primers (Table 1) were designed based on the transcript sequences obtained from the RNA-seq data using Primer Premier v5 software. Primers were synthesized by Sangon Biotech Co., Ltd. (Shanghai, China) and diluted to a working concentration of 10 μM. The qRT-PCR assays were performed in a 20-μL reaction volume using the ChamQ Universal SYBR qPCR Master Mix (Catalog No. Q711; Vazyme Biotech Co., Ltd., Nanjing, China) on a CFX96 Real-Time PCR Detection System (Bio-Rad, Hercules, CA, USA). Each reaction mixture contained 10 μL of 2× ChamQ Universal SYBR qPCR Master Mix, 0.5 μL of each forward and reverse primer (10 μM), 2 μL of cDNA template, and RNase-free water to reach a final volume of 20 μL. The amplification cycling conditions consisted of an initial denaturation step at 95 °C for 30 s, followed by 38 cycles of denaturation at 95 °C for 10 s, annealing at 55 °C for 30 s, and extension at 72 °C for 10 s. A subsequent melting curve analysis (95 °C for 15 s, 60 °C for 1 min, and 95 °C for 15 s) was conducted to confirm the amplification specificity. The relative expression levels of the target genes were calculated using the 2^−ΔΔ*Ct*^ method, with the 60S ribosomal protein RPL13 (UniProt accession: Q962U1) serving as the internal reference gene for normalization. The calculation equation used was: ΔΔ*Ct* = Δ*Ct*_cold treatment_ − Δ*Ct*_control_. All assays were performed with three independent biological replicates, and each biological replicate included three technical replicates.

### 2.6. Statistical Analysis

All statistical analyses were performed using SPSS version 23.0 (IBM Corp., Armonk, NY, USA). Prior to analysis, the data were tested for normality and homogeneity of variance using the Shapiro–Wilk test and Levene’s test, respectively. Differences in larval mandible width, survival rates, SCPs, body water content, and relative gene expression levels across different treatments were analyzed using one-way analysis of variance (ANOVA). When significant differences were detected, Tukey’s HSD test was applied for multiple comparisons between groups. All data are presented as the mean ± standard error (*SE*), and statistical significance was defined at *p* < 0.05.

## 3. Results

### 3.1. Relationship Between Larval Development and Environmental Temperature

**Figure 1:**
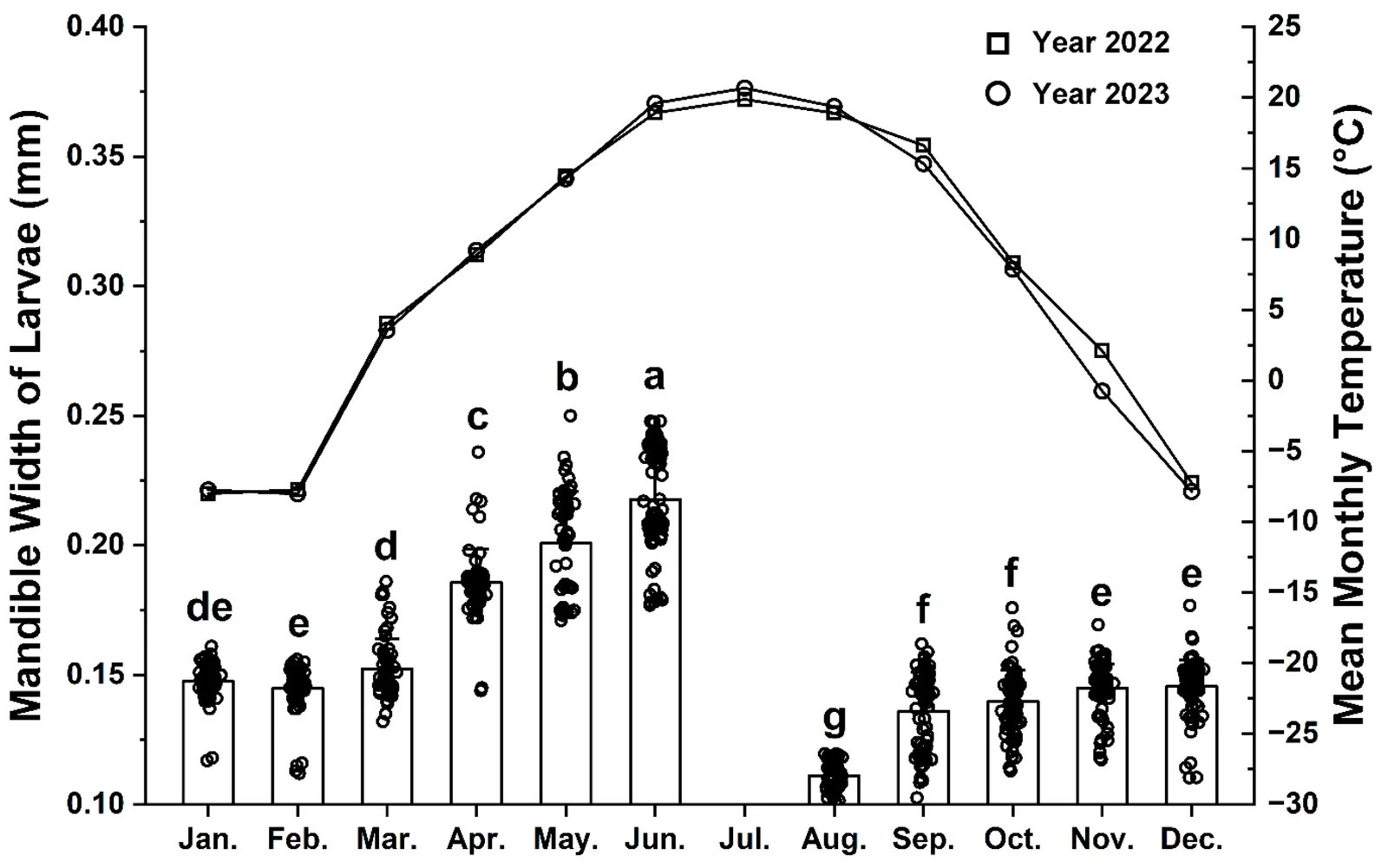
Mandible width of *M. sabinae* larvae and monthly mean habitat temperatures (2022–2023). The left Y-axis represents the larval mandible width, serving as an indicator of the developmental stage, while the right Y-axis represents the monthly mean temperature. Mandible width data are presented as the mean ± *SE*. Different lowercase letters above the error bars indicate significant differences among months (Tukey’s HSD test, *p* < 0.05; *n* = 60).

Monthly mean temperature significantly influenced the larval development of *M. sabinae* (Figure 1). After hatching, the larvae underwent an initial growth phase from August to November. Subsequently, they entered a period of developmental arrest from November to February of the following year, driven by the low ambient temperatures. From March to June, the larvae resumed growth and development until pupation. Throughout the overwintering period, the lowest monthly mean temperature of the habitat remained above −10 °C. Notably, larval developmental arrest was initiated once the monthly mean temperature dropped below 5 °C.

### 3.2. Effect of Low-Temperature Duration on the Survival Rate of M. sabinae Larvae

**Figure 2:**
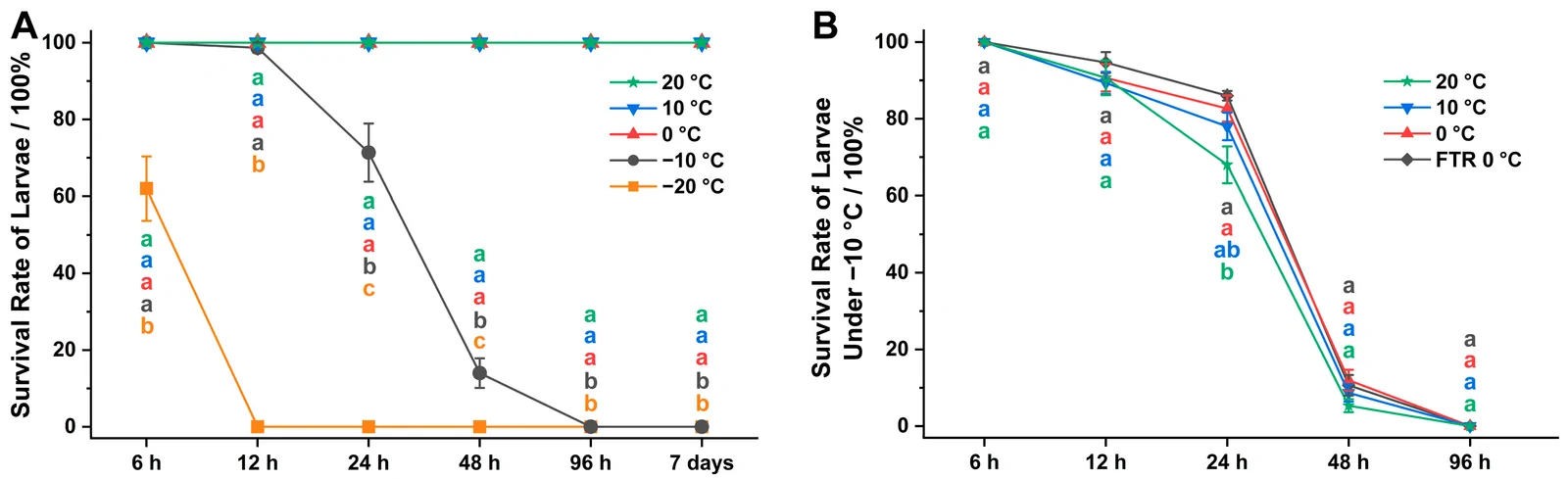
Survival rates of *M. sabinae* larvae following low-temperature treatments. (**A**) Survival rates of *M. sabinae* larvae across six exposure durations under five constant temperature treatments (20 °C, 10 °C, 0 °C, −10 °C, and −20 °C). (**B**) Larval survival at five time points during exposure to −10 °C, following a 7 d cold preconditioning period under various regimes. Survival rate data are presented as mean ± *SE*. Different lowercase letters indicate significant differences among the different temperature treatments at the same exposure duration (Tukey’s HSD test, *p* < 0.05; *n* = 5).

Low-temperature treatments significantly influenced the survival rate of *M. sabinae* larvae (Figure 2). Survival assays across various constant temperatures revealed that no larval mortality occurred at 0 °C, 10 °C, and 20 °C over the 7 d exposure period. At −10 °C, mortality was first observed after 6 h of exposure, whereas exposure to −20 °C resulted in high mortality within 6 h (Figure 2A). These findings indicate that larvae can survive normally at temperatures between 0 °C and 20 °C, whereas exposure to −10 °C and lower temperatures leads to rapid mortality. Furthermore, evaluating the effect of cold acclimation on survival at −10 °C demonstrated that preconditioning at 0 °C (either under constant conditions or a fluctuating thermal regime) significantly improved larval survival rates following 24 h of exposure to −10 °C (Figure 2B).

### 3.3. Effect of Cold Preconditioning on the Supercooling Point and Body Water Content of M. sabinae Larvae

**Figure 3:**
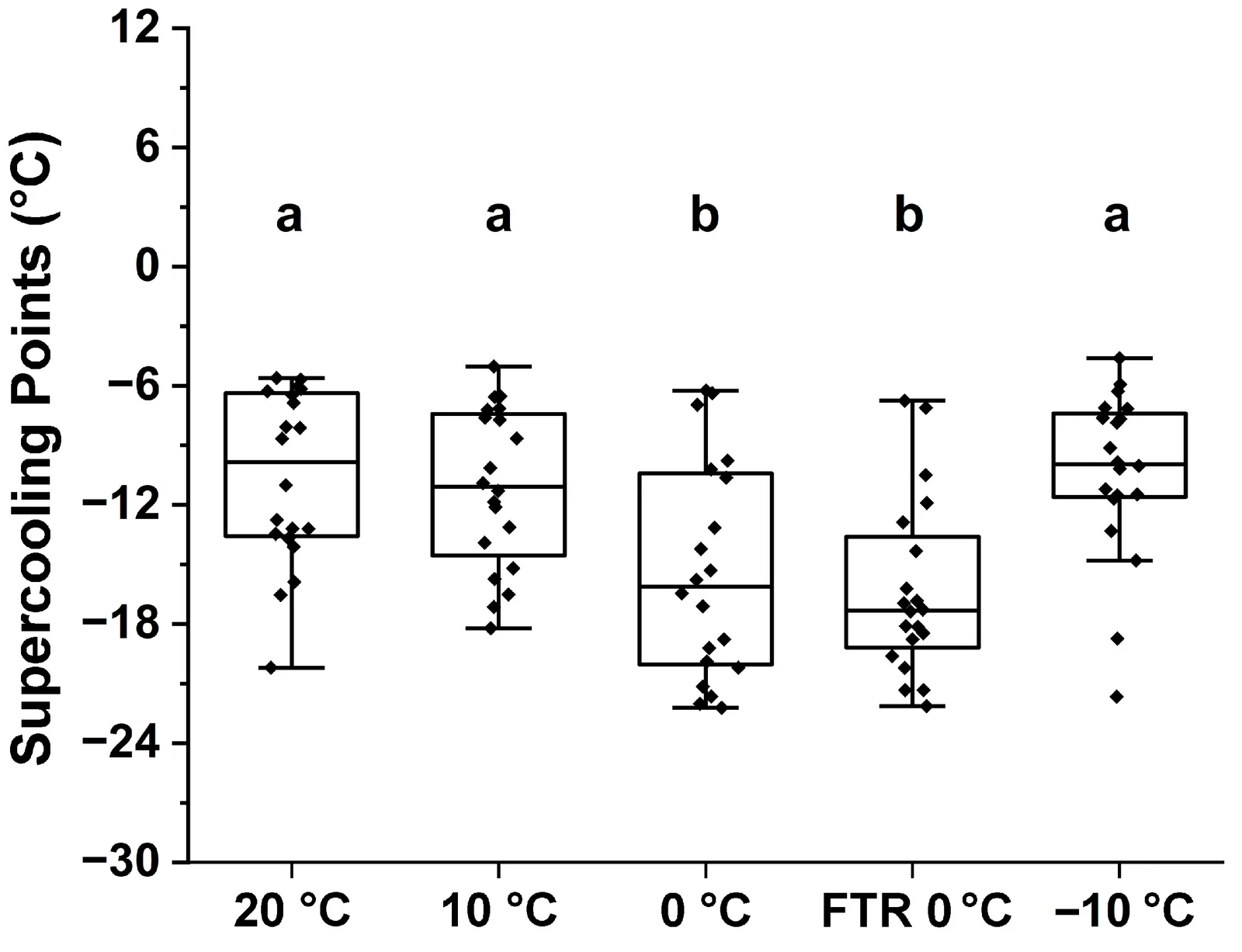
Supercooling points of *M. sabinae* larvae following a 7 d preconditioning period at different temperatures. Data are presented as boxplots with overlaid jittered points representing individual raw data values. For each boxplot, the horizontal line within the box represents the median, and the lower and upper boundaries of the box indicate the 25th and 75th percentiles, respectively. The whiskers extend to the minimum and maximum values within 1.5 times the interquartile range (IQR). Different lowercase letters indicate significant differences among the different preconditioning treatments (Tukey’s HSD test, *p* < 0.05; *n* = 20).

No significant differences in the SCP were observed among the 20 °C, 10 °C, and −10 °C preconditioning groups. In contrast, preconditioning at 0 °C and under the fluctuating thermal regime (FTR 0 °C) significantly depressed the SCP of *M. sabinae* larvae (Figure 3).

Furthermore, we detected no significant differences in larval free water content across the five preconditioning treatments (Table 2).

### 3.4. Identification of AQP and Neuropeptide Capa Receptor Genes in M. sabinae

A total of 17 AQP-like and 1 neuropeptide *Capa* receptor genes were identified in *M. sabinae* (Table 3). Among the AQPs, four genes shared 38.1–55.1% sequence identity with homologous genes from other hymenopteran species. Of the 17 AQP-like genes, only three were significantly differentially expressed under the 0 °C cold treatment compared to the 20 °C control (two upregulated and one downregulated), while the remaining genes showed no significant changes (Table 3). Among these three differentially expressed genes, DN27260_c0_g1 exhibited the highest sequence identity (69.5%) to *TIP1-1* of *Trichinella patagoniensis*; DN5722_c0_g1 shared 55.1% identity with *TSAR_002350* of *Trichomalopsis sarcophagae*; and DN36733_c0_g1 had the lowest identity (52.1%) with an aquaporin-like protein from *Pieris brassicae*. Expression of the only neuropeptide *Capa* receptor gene (sharing 46.7% identity with a *Ceratosolen solmsi Capa* receptor-like gene) was not significantly affected by cold exposure.

### 3.5. Expression Profiles of AQP-Related Genes Under Low-Temperature Exposure

**Figure 4:**
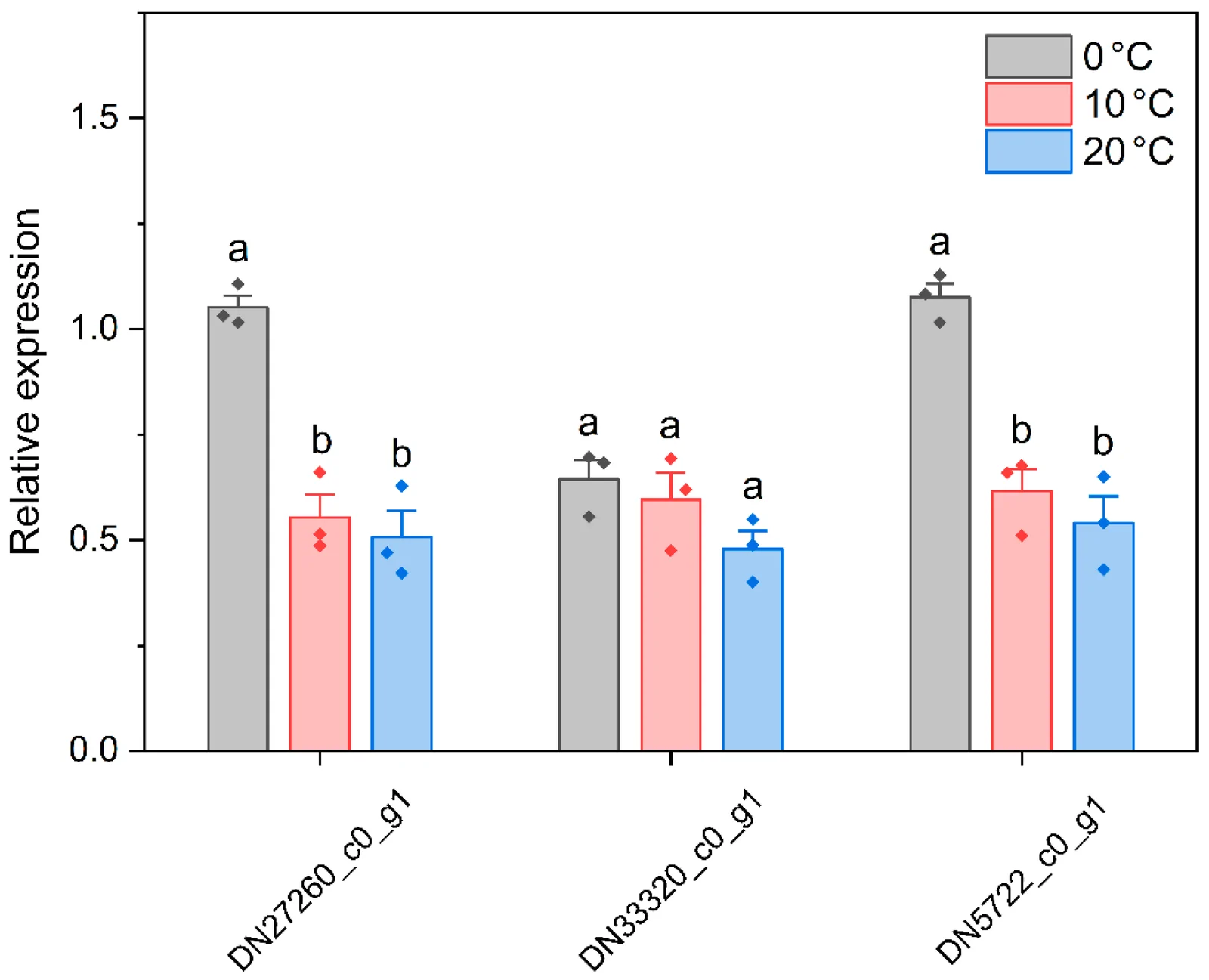
Relative mRNA expression levels of AQP-like genes in *M. sabinae* larvae under different temperature treatments. Relative expression levels were normalized using *RPL13* as the internal reference gene. Data are presented as the mean ± *SE* (*n* = 3 biological replicates). Different lowercase letters indicate significant differences among the temperature treatments (20 °C, 10 °C, 0 °C) for each respective gene (Tukey’s HSD test, *p* < 0.05).

To validate the accuracy of the RNA-seq data, we selected three upregulated AQP-like genes for quantitative real-time PCR (qRT-PCR) analysis. The expression patterns obtained from qRT-PCR were highly consistent with the transcriptomic results (Table 3, Figure 4). Following a 7 d cold treatment, the relative mRNA expression levels of DN27260_c0_g1 (*TIP-1*) and DN5722_c0_g1 (*AQPN*) were significantly upregulated at 0 °C compared to those at 10 °C and the 20 °C control. Conversely, while the expression of DN33320_c0_g1 (*TIP1-2*) exhibited an upward trend at 0 °C and 10 °C relative to 20 °C, these changes were not statistically significant (Figure 4).

## 4. Discussion

Investigating the cold tolerance and overwintering mechanisms of high-altitude insects is fundamental to understanding their environmental adaptation. To our knowledge, this study provides the first systematic elucidation of the low-temperature adaptation strategies in phytophagous wasp larvae. Specifically, larval growth and development cease when the monthly mean habitat temperature falls below 5 °C. Furthermore, laboratory cold acclimation significantly enhances larval survival under extreme low temperatures at 24 h and markedly depresses the supercooling point (SCP). At the molecular level, cold stress triggers the transcriptional responses of a few of the many existing aquaporin (AQP) genes. Interestingly, *M. sabinae* larvae do not exhibit significant changes in free body water content in response to low temperatures, suggesting that the cold-induced differential expression of AQP-related genes may constitute only a fraction of their broader cold tolerance mechanism.

Dormancy is a crucial overwintering strategy in insects and is primarily classified into two types: quiescence and diapause [29]. Our laboratory observations revealed that when overwintering *M. sabinae* larvae were transferred from prolonged low-temperature conditions to room temperature, they rapidly resumed activity and continued normal development within a few hours (unpublished data). Therefore, the overwintering dormancy of *M. sabinae* larvae represents quiescence rather than diapause. This mechanism is consistent with findings in other insect species; for instance, adults of the weevil *Eucryptorrhynchus brandti* Harold resume reproductive development immediately upon transfer from cold field conditions to the laboratory [30]. Similarly, larvae of the pistachio twig borer, *Kermania pistaciella* Amsel., cease development and enter a quiescent overwintering state solely in response to autumn temperatures dropping below their developmental threshold [31].

Unlike diapause, which is a genetically programmed state typically accompanied by anticipatory and profound physiological remodeling prior to the onset of winter, quiescence is primarily a direct, passive physiological response to declining ambient temperatures [32]. Because quiescent insects do not typically undergo massive anticipatory cold hardening well in advance of thermal stress, their basal physiological defenses—prior to sufficient cold acclimation—can be somewhat limited [33]. Furthermore, rather than migrating to heavily insulated overwintering refuges such as soil or deep leaf litter, *M. sabinae* larvae overwinter in situ within the cones of the host plant canopy. Overwintering in such an exposed above-ground habitat forces these quiescent larvae to confront more extreme and fluctuating meteorological conditions than ground-dwelling insects [34,35].

The basal cold tolerance of the larvae is relatively limited: constant temperature exposure at −20 °C resulted in high mortality within 6 h (Figure 2A), classifying them as freeze-avoidant or chill-susceptible insects [36]. Notably, although the lowest monthly mean air temperature only dropped to approximately −10 °C, the daily minimum temperature could fall below −20 °C (unpublished data)—a temperature lower than the SCP of several larvae evaluated in this study (Figure 3). However, subsequent dissections of cones from the same sampling site the following spring revealed negligible freezing-induced mortality (unpublished data). This suggests that *M. sabinae* larvae in the field do not experience these extreme ambient minimums directly; rather, they avoid lethal freezing through the thermal buffering effect of the cone microhabitat. In our laboratory assays, cold treatments were applied to excised cone-bearing branches, which cannot fully replicate the microclimate of intact *Juniperus* plants in the field [37]. The internal temperature of alpine plant tissues often deviates from the ambient air temperature, typically exhibiting more gradual fluctuations. Therefore, relying solely on ambient air temperatures to estimate the thermal stress experienced by the insects can introduce significant bias. Consequently, accurately assessing field cold tolerance and overwintering dynamics requires monitoring or simulating the actual microclimate temperatures within the plant tissues.

Both constant 0 °C and fluctuating thermal regime (FTR) pretreatments conferred a slight, albeit statistically significant, increase in the survival of *M. sabinae* larvae under the extreme low temperature of −10 °C (Figure 2B). While this pattern demonstrates a baseline cold acclimation (CA) response, whereby insects enhance their basal cold tolerance following a sustained exposure to non-lethal cold, the marginal practical improvement indicates that acclimation alone is insufficient to provide robust protection against prolonged extreme freezing. Unlike rapid cold hardening (RCH) [38], which occurs within minutes to hours, the 7 d acclimation period employed in this study provides sufficient time for the mobilization of physiological and molecular defenses. Although the absolute survival benefit was modest, such phenotypic plasticity has been widely documented across various insect taxa, underscoring its ecological relevance [39,40,41,42,43]. Moreover, the FTR applied in this study simulated natural diel temperature fluctuations (a −5 °C/0 °C/5 °C cycle); the cold hardiness it induced was comparable to, and at some time points even slightly stronger than, that produced by a constant 0 °C exposure (Figure 2B). Under natural conditions, alpine regions experience dramatic diel temperature oscillations during the autumn-winter transition. Such frequent thermal variation, acting over a period of several days, may serve as a reliable seasonal cue that triggers the progressive accumulation of cryoprotectants and the activation of protective pathways. Compared with constant temperatures, a fluctuating thermal signal more faithfully reproduces the gradual natural cooling process and therefore carries greater ecological significance as a cue for seasonal cold adaptation [44,45]. For example, larvae of *Eurosta solidaginis* Fitch subjected to simulated diel temperature fluctuations accumulated significantly more glycerol and exhibited greater cold tolerance than those held at constant temperatures [46]. Thus, the protective physiological and biochemical changes elicited during the 7 d acclimation period constitute the intrinsic basis for the subsequent improvement in survival [47].

The supercooling point (SCP) is a key indicator of cold hardiness in freeze-avoidant insects, representing the lowest temperature at which body fluids can remain in a supercooled state in the absence of ice nucleation [48,49]. In this study, both constant 0 °C and the fluctuating thermal regime (FTR) significantly depressed the SCP of *M. sabinae* larvae (Figure 3). A critical physiological mechanism by which cold acclimation enhances cold tolerance is through this precise reduction in the SCP. Previous studies have shown that cold acclimation can promote the accumulation of low-molecular-weight cryoprotectants (e.g., glycerol and sorbitol) or upregulate the expression of heat shock proteins; these factors collectively stabilize the supercooled state and delay ice nucleation. Consequently, a depressed SCP minimizes the risk of spontaneous freezing at sub-zero temperatures, which directly accounts for the enhanced survival of acclimated larvae in a −10 °C environment. Traditionally, the major mechanisms for lowering the SCP are thought to include the evacuation of gut contents to eliminate ice-nucleating agents, the reduction in free body water (dehydration), and the accumulation of cryoprotective solutes such as glycerol and trehalose [8,48]. However, our measurements revealed no significant differences in free body water content among larvae across the five acclimation treatments (Table 2). This intriguing phenomenon—a depressed SCP without concurrent dehydration—clearly rules out simple body water loss as the primary driver of SCP reduction in *M. sabinae*, suggesting that their overwintering strategy is largely dehydration-independent and must be mediated by alternative biochemical pathways.

Given the absence of dehydration, we propose that the observed SCP depression is primarily driven by the active, de novo synthesis and massive accumulation of low-molecular-weight cryoprotectants within the larvae. Typically, freeze-avoidant insects rely on significant body water loss to passively concentrate internal solutes. Because *M. sabinae* larvae retain a high free water content during cold acclimation, they face a unique biophysical challenge. To achieve the observed ~6 °C depression in SCP without dehydration, they must allocate considerable metabolic resources to synthesize specific low-molecular-weight solutes (such as polyols, sugars, and free amino acids) at sufficiently high concentrations to alter the colligative properties of their fully hydrated body fluids [50]. Furthermore, beyond purely colligative freezing point depression, these compounds are indispensable for non-colligatively stabilizing cellular structures under severe alpine cold stress. The massive accumulation of such compatible solutes is critical to preserve phospholipid bilayer integrity, prevent lethal membrane phase transitions, and stabilize native protein conformations, thereby maintaining cellular homeostasis even at subzero temperatures [35,51]. While most insect species synthesize a single dominant polyol, some employ a multi-component system, with glycerol and sorbitol representing the most prevalent combination [52,53]. In several freeze-avoidant insects, such as the goldenrod gall moth *Epiblema scudderiana* Clemens and the European corn borer *Ostrinia nubilalis* Hübner, seasonal elevations in glycerol concentration are strongly correlated with SCP depression [54,55]. Furthermore, the accumulation of antifreeze proteins (AFPs) and other non-colligative cryoprotectants may act synergistically to further depress the SCP, although the synthesis of such macromolecules generally requires prolonged acclimation periods [9]. Future studies should employ metabolomic approaches to systematically quantify changes in the profiles of cryoprotectants—such as glycerol, trehalose, and sorbitol—in *M. sabinae* larvae before and after cold acclimation. Additionally, incorporating assessments of ice-nucleating activity will be essential to fully elucidate the exact biochemical mechanisms underlying this dehydration-independent SCP depression [49].

Despite these laboratory observations characterizing *M. sabinae* as a species with limited basal cold tolerance (e.g., high mortality during prolonged exposure to −10 °C), there remains uncertainty regarding its exact cold-hardiness strategy in nature. When overwintering inside the well-insulated microhabitat of the cones, the larvae might experience a slow cooling process at relatively high sub-zero temperatures. Under such buffered conditions, they could potentially undergo gradual extracellular freezing and become freeze-tolerant to some extent. If this occurs, the supercooling point (SCP) would not be the sole or most relevant ecological indicator of their winter survival limit. The physiological dynamics of slow extracellular freezing are classically exemplified by the goldenrod gall fly, *E. solidaginis* [24,46]. In *E. solidaginis*, extracellular freezing is a gradual process where water is drawn out of cells into growing ice crystals, increasing intracellular solute concentrations until an osmotic equilibrium is reached [46]. While *M. sabinae* clearly lacks the extreme cold-hardiness capacity of *E. solidaginis*—which can survive exposure to temperatures as low as −80 °C and endure maximal ice formation of approximately 65% of its total body water—it may rely on fundamentally similar metabolic shifts to survive milder, temporary extracellular freezing in the field, such as drawing upon anaerobic glycolysis to supply basal energy demands.

Given that the free water content remained consistent across all acclimation treatments (Table 2), we further examined the expression profiles of genes involved in water regulation using the transcriptomic dataset. Our analysis focused on the aquaporin (AQP) gene family and the neuropeptide *Capa* receptor gene, which is a known modulator of diuresis in insect Malpighian tubules [27,56]. The results indicated that after 7 d of acclimation at 0 °C, the expression levels of the vast majority of identified AQP-related genes (e.g., DN16314, DN3440, and DN49776) remained unchanged, and the neuropeptide *Capa* receptor gene (DN1757_c0_g2) likewise showed no significant differential expression (Table 3). These molecular profiles are highly consistent with the stable free water content observed physiologically, suggesting that cold acclimation at 0 °C does not activate the water excretion pathway in *M. sabinae* larvae.

Nevertheless, a small subset of AQP genes was significantly upregulated under low-temperature conditions, specifically DN27260_c0_g1 (annotated as TIP1-1) and DN5722_c0_g1 (annotated as AQPN). Relying solely on sequence homology, however, makes it difficult to definitively assign specific functions to these upregulated candidates. Systematic functional validation is therefore imperative for future studies. First, the substrate permeability (e.g., water, glycerol, sorbitol, and urea) of these AQPs should be empirically determined using heterologous expression systems, such as *Xenopus oocytes* Daudin or *Saccharomyces cerevisiae* Meyen ex E.C. Hansen [57,58]. Second, RNA interference (RNAi) or CRISPR/Cas9-mediated gene knockout should be employed to assess the phenotypic consequences in *M. sabinae* larvae [59]. Examining changes in SCP, free water content, cryoprotectant concentrations, and survival rates following cold acclimation in these gene-silenced individuals will allow us to determine the functional contribution of these specific AQPs to cold hardiness at the organismal level.

## 5. Conclusions

In summary, our laboratory assessments indicate that *M. sabinae* larvae utilize a freeze-avoidant overwintering strategy, relying on cold acclimation to significantly depress their SCP without an overall reduction in free body water content. This dehydration-independent mechanism is corroborated at the molecular level by the lack of differential expression in the vast majority of aquaporin genes, with transcriptional adjustments strictly limited to specific channels (TIP1-1 and AQPN). However, their overwintering strategy in the natural cone microhabitat may be more complex, potentially involving slow-cooling-induced freeze tolerance. Future work should integrate metabolomic, proteomic, and functional genomic approaches to comprehensively elucidate both the dehydration-independent cold hardiness mechanisms and the potential for extracellular freezing survival in *M. sabinae* larvae.

## Figures and Tables

**Table 1:** Primer sequences used for qRT-PCR validation.

Gene ID	Gene Name	Primer Sequence (5′-3′)	Product Size (bp)
DN27260_c0_g1	TIP1-1	F: ACTGTCTCATGGCATAGGCG	175
		R: GACCGCTCTACACACAACCA	
DN33320_c0_g1	TIP1-2	F: CACCGTGTGCCACCTTCAAC	154
		R: GTGACATTTGGCGCTCTTGT	
DN5722_c0_g1	AQPN	F: TTGAGAGCGGCACTACCAGC	169
		R: AACCCTTGCAATCCTCACGT	

**Table 2:** Body water content after different temperature cold preconditioning.

Preconditioning Temperature	Fresh Weight (mg)	Dry Weight (mg)	Free Water Content (100%)
20 °C	104.7 ± 4.9 a	29.4 ± 3.7 a	72.2 ± 2.3 a
10 °C	101.7 ± 8.1 a	25.7 ± 4.1 a	74.9 ± 3.3 a
0 °C	89.4 ± 6.8 a	28.2 ± 2.6 a	68.5 ± 1.2 a
FTR 0 °C	95.3 ± 8.3 a	32.5 ± 3.4 a	65.5 ± 3.4 a
−10 °C	111.3 ± 10.6 a	33.1 ± 3.3 a	70.3 ± 1.1 a

Note: Body water content shows a batch of 100 *M. sabinae* larvae following a 7-day cold preconditioning. Lowercase letters in the same column indicate differences among the different temperature treatments. Data are shown as the mean ± *SE* (Tukey’s HSD test, *p* < 0.05, *n* = 4).

**Table 3:** Differentially expressed AQPs and neuropeptide *Capa* receptor genes in *M. sabinae* under different temperature treatments.

Category	Gene ID	Gene Name	NR Description (Species)	Accession No.	Identity (%)	0 vs. 20		10 vs. 20	
						Log_2_fc	*P_adj_*	Log_2_fc	*P_adj_*
Aquaporins	DN16314_c0_g1	TIP1-2	putative aquaporin TIP1-1 (*Trichinella patagoniensis*)	P0DO54	73.8	6.4868	1.0000	1.1747	1.0000
	DN18837_c0_g1	PIP1-3	putative aquaporin PIP1-2 (*Trichinella patagoniensis*)	Q08733	91.5	2.4793	0.6422	−0.2899	1.0000
	DN20240_c0_g1	AQPN	predicted: aquaporin isoform X2 (*Ceratosolen solmsi marchali*)	Q25074	44.2	0.2138	0.8068	0.2455	0.6201
	DN27260_c0_g1	TIP1-1	putative aquaporin TIP1-1 (*Trichinella patagoniensis*)	P25818	69.5	4.4361	0.0199 *	1.1945	1.0000
	DN33320_c0_g1	TIP1-2	predicted: aquaporin-4-like (*Branchiostoma belcheri*)	Q94CS9	61	4.0400	0.1488	1.6545	1.0000
	DN3440_c0_g1	PIP1-4	putative aquaporin PIP1-2 (*Trichinella patagoniensis*)	Q39196	87	4.8382	1.0000	−1.1590	0.7045
	DN36733_c0_g1	AQP	aquaporin-like (*Pieris brassicae*)	C4VBN2	52.1	−3.3129	0.0004 *	−4.0396	0.0000
	DN49776_c0_g1	PIP2-8	putative aquaporin PIP1-2 (*Trichinella patagoniensis*)	Q9ZVX8	90.9	4.4939	1.0000	0.0000	1.0000
	DN54152_c0_g1	TIP-type	putative aquaporin TIP1-1 (*Trichinella patagoniensis*)	P42067	80.2	4.0714	1.0000	0.0000	1.0000
	DN5480_c0_g1	AQPN	aquaporin AQPcic-like (*Copidosoma floridanum*)	Q25074	38.1	−0.2223	0.6640	−0.2143	0.6309
	DN54993_c0_g1	TIP1-1	aquaporin AQPAe.a-like isoform X2 (*Copidosoma floridanum*)	P50156	50.8	−3.4082	1.0000	−3.5100	1.0000
	DN5722_c0_g1	AQPN	hypothetical protein TSAR_002350 (*Trichomalopsis sarcophagae*)	Q7PWV1	55.1	1.9103	0.0451 *	1.0812	0.2908
	DN76309_c0_g1	NIP2-2	aquaporin NIP2-2 (*Geodia barretti*)	Q67WJ8	94.9	−2.6117	1.0000	−2.6980	1.0000
	DN76517_c0_g1	AQP1	aquaporin-4-like (*Saccoglossus kowalevskii*)	Q02013	100	6.8099	1.0000	0.0000	1.0000
	DN77799_c0_g1	AQP3	protein product (*Bursaphelenchus okinawaensis*)	Q92482	52.2	5.9679	1.0000	0.0000	1.0000
	DN80393_c0_g1	PIP2-6	putative aquaporin PIP2-6 (*Trichinella patagoniensis*)	Q7XLR1	65.2	5.2400	0.2605	0.0000	1.0000
	DN86115_c0_g1	AQP5	predicted: aquaporin-4-like (*Saccoglossus kowalevskii*)	Q9WTY4	100	5.3111	1.0000	0.0000	1.0000
*Capa* receptor gene	DN1757_c0_g2	Cap2bR	neuropeptides *Capa* receptor-like (*Ceratosolen solmsi marchali*)	Q8ITC7	46.7	−0.1043	0.7693	−0.4039	0.1379

Note: *P_adj_* represents the *p*-value adjusted by the Benjamini–Hochberg method. Genes with *P_adj_* < 0.05 were considered significantly differentially expressed, and an asterisk (*) indicates a significant difference.

## Data Availability

The original contributions presented in this study are included in the article. Further inquiries can be directed to the corresponding author.
